# High‐Intensity Interval Training Boosts Immune Cells in Advanced Cancer Patients and Healthy Controls: Implications for Cancer Care

**DOI:** 10.1002/cam4.71977

**Published:** 2026-06-03

**Authors:** Fabian Kiehl, Nico De Lazzari, Ronja Beller, Isabella Deppe, Sabrina B. Bennstein, Suzan Botzenhardt, Maria Rosa Salvador Comino, Mitra Tewes, Miriam Götte

**Affiliations:** ^1^ Department of Palliative Medicine University Hospital Essen, University of Duisburg‐Essen Essen Germany; ^2^ Institute for Movement and Exercise West German Cancer Center, University Hospital Essen Essen Germany; ^3^ Department of Pediatric Hematology/Oncology Clinic for Pediatrics 3, University Hospital Essen, University of Duisburg‐Essen Essen Germany; ^4^ Institute of Immunology, Medical Faculty RWTH Aachen University Aachen Germany

**Keywords:** advanced cancer patients, circulating innate lymphoid cells, natural killer cells, palliative care, physical activity

## Abstract

**Background:**

Regular exercise improves quality of life (QoL) and reduces fatigue in advanced cancer patients (ACP), but acute immune responses are underexplored. This study investigated the effects of a single high‐intensity interval training (HIIT) session on natural killer (NK) cells and circulating innate lymphoid cells (cILCs) in ACP.

**Methods:**

An ACP group undergoing chemotherapy (PG, *n* = 20, 59.8 ± 10.3 years, 70% pancreatic cancer) was compared with age‐matched healthy controls (HG, *n* = 19, 60.7 ± 10.6 years). Both groups completed a 20‐min HIIT session. Blood samples were collected before (T0), immediately after (T1), and 1 h post‐exercise (T2). NK cells, cILCs, and subsets were analyzed via flow cytometry. Patient‐reported outcomes (QoL, fatigue, nutrition) were assessed with validated questionnaires.

**Results:**

A single HIIT session induced a significant (PG: *p* = 0.001; HG: *p* = 0.045) increase in the number of circulating total NK cells immediately after exercise in both groups, with a median within‐subject increase of 50% in the PG and 38% in the HG, followed by a significant (PG/HG: *p* < 0.001) decrease during the recovery period (PG: –43%, HG: −69%). cILCs increased significantly (PG: *p* = 0.005; HG: *p* = 0.001) only during the exercise period in both groups, with a median within‐subject increase of 21% in the PG and 55% in the HG. Differences were observed between groups and across individual immune cell subpopulations. NK responses positively correlated with higher training heart rate in both groups, and additionally with BMI (HG, cILC2) and age (PG, CD56^bright^NK cells).

**Conclusion:**

A single HIIT session transiently mobilized NK cells and cILCs in ACP undergoing chemotherapy. These results indicate that acute HIIT is feasible in this population and provide hypothesis‐generating evidence on exercise‐induced changes in immune cell counts.

**Trial Registration:**

The study was registered at 13.11.2022, registration number NCT05656651

**Abbreviations**
ACP
: Advanced cancer patients
BMI
: Body mass index
cILCs
: Circulating innate lymphoid cells
HG
: Healthy control group
HIIT
: High‐intensity interval training
HR
: Heart rate
NK cells: Natural killer cells
PG
: Patient group
RPE
: Rate of perceived exertion

## 1 Introduction

The World Health Organization reports that cancer was the second leading cause of death, with 9.6 million deaths in 2018 [1]. The incidence and mortality of cancer are expected to increase due to demographic changes including population aging and growth as well as increasing life expectancy [2, 3]. The increase in cancer incidence is accompanied by an increased need for palliative care, with 56.8 million patients currently receiving palliative care worldwide [4]. As disease progresses, improving quality of life (QoL) and controlling symptoms such as pain, fatigue, cachexia, weakness, and dyspnea [5] become crucial. Advanced cancer patients (ACP) undergoing palliative treatment often experience systemic inflammation, which is linked to lower overall survival [6] and higher overall Eastern Cooperative Oncology Group (ECOG) performance status [7, 8]. Natural killer (NK) cells and circulating innate lymphoid cells (cILCs), as fast‐acting immune cells, are thought to have anti‐inflammatory effects [9], suggesting that exercise may represent a promising approach to address the aforementioned symptoms. Positive effects of long‐term physical activity interventions have been demonstrated, for example, in lung cancer [10] and colorectal cancer [11]. These effects include improvements in physical functioning and health‐related QoL of ACP [12, 13, 14], as well as beneficial changes to the immune system [15]. Exercise has been shown to enhance the infiltration of immune cells, including NK cells, into the tumor microenvironment [16]. However, immune responses to exercise depend on exercise characteristics such as duration, as prolonged cortisol exposure may impair T‐cell effector functions [17].

CD56^+^NK cells, which are part of the innate immune system, target pathogenic and tumor cells by detecting missing major histocompatibility complex class I molecules, triggering apoptosis [18]. They are classified as CD56^dim^ (mainly cytotoxic through antibody‐dependent cell‐mediated cytotoxicity) or CD56^bright^ (mainly cytokine‐producing) NK cells [9] on the basis of their CD56 expression. CD56^dim^NK cells, via CD16, bind immunoglobulins to release IFN‐γ and cytotoxic substances that cause target cell lysis and apoptosis [19], making the CD16^+^CD56^dim^NK cell count a measure of functionality. Exercise‐driven NK cell mobilization into the circulation has been demonstrated in both healthy individuals [20, 21] and cancer patients [22, 23]. Several studies in patients with curative cancer have reported increased numbers of circulating NK cells after a single exercise bout [24, 25], however, data concerning ACP remain limited.

Innate lymphoid cells (ILCs), including ILC1, ILC2, and ILC3 [26], exist as tissue‐resident (tILCs) and circulating ILCs (cILCs), with most ILCs in tissues and a small proportion in circulation [23]. cILC1 are NK cell precursors [27, 28], cILC2 contribute to allergic reactions [29] and defense against parasites [30], while cILC3 can be divided into NKp44^+^ (which is expressed in various autoimmune diseases [31], inflammatory bowel diseases [32] and leukemia [33]) and NKp44^−^ subgroups. Recent evidence has further expanded the functional relevance of ILC subsets in tumor immunity and immune surveillance. In line with their role as NK cell precursors [27, 28] ILC1 have been shown to give rise to mature KIR^+^NK cells [27, 34], thereby directly linking this subset to the development of cytotoxic effector functions. Moreover, ILC2 have been reported to mediate granzyme B‐dependent tumor cell killing [35], further supporting a potential contribution of ILC subsets to anti‐tumor immunity. Despite these emerging insights, the exercise responsiveness of circulating ILC subsets, particularly in ACP, remains largely unexplored.

Given the evidence of exercise‐induced immune cell mobilization and the relevance of NK cells and cILCs for anti‐inflammatory effects and QoL, we hypothesized that a single 20‐min high‐intensity interval training (HIIT) session would induce acute changes in circulating immune parameters in ACP and matched healthy controls, with comparable responses between groups.

## 2 Methods

### 2.1 Study Design and Recruitment

**FIGURE 1:**
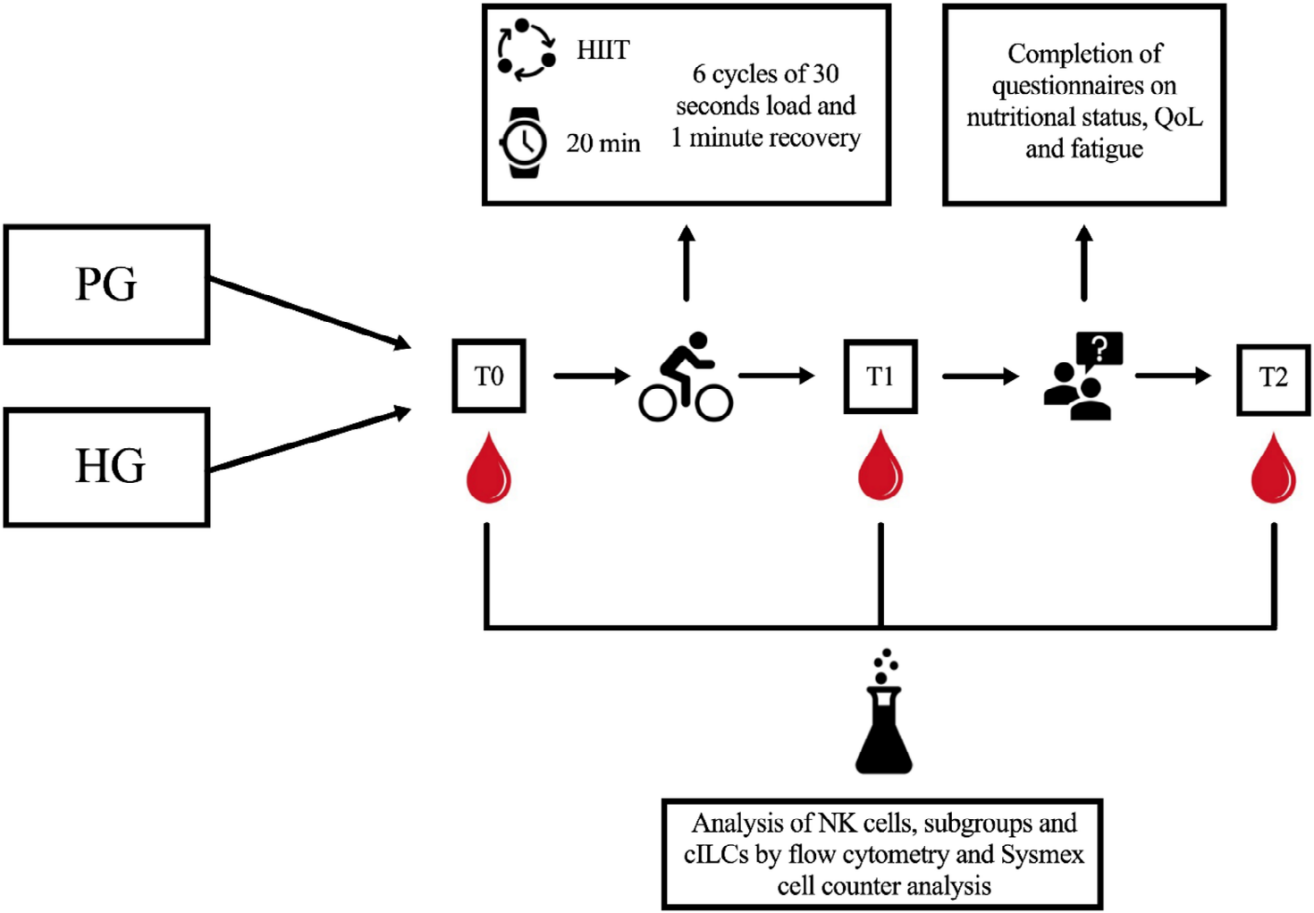
Study design with PG and HG, blood samples taken at each time point (T0, T1, T2), exercise intervention on a bicycle ergometer, completion of questionnaires during the recovery period and subsequent laboratory analysis.

This monocentric interventional trial included an ACP group (PG) undergoing chemotherapy and a healthy control group (HG). Patients were screened between November 2022 and July 2023 at a university cancer hospital. Written informed consent was obtained prior to inclusion. The HG was recruited until July 2024 to match the PG in terms of sex, age (±5 years), BMI (±5 kg/m^2^), and exercise interest (±3 points, scale 1 = not interest; 10 = very high interest). Subjects were deemed ineligible for the control group if they had serious chronic or other illnesses, were pregnant or had other contraindications to intense physical activity as certified by a physician. Suitable control subjects were recruited through responses to informal flyers as well as through active outreach conducted in outpatient clinics, community organizations, sports clubs, and patients' broader social networks and circles of acquaintances. The study was registered at ClinicalTrials.gov (NCT05656651) and approved by the Ethics Committee of the Medical Faculty Duisburg‐Essen (approval number: 19‐8789‐BO). Blood samples were collected before (T0), immediately after (T1), and one hour after HIIT (T2). The primary aim was to analyze the immune response to HIIT in the PG and matched HG (see Figure 1) from T0 to T1, with a focus on NK cells and cILCs. This study was conducted as an exploratory investigation of acute immune responses to a single HIIT session in ACP and matched healthy controls. No formal power calculation was performed. NK cells were defined as the primary endpoint given their established responsiveness to acute exercise, and cILCs were included as exploratory endpoints to provide initial insights into these less‐studied populations. Secondary endpoints included changes in blood cell counts from T1 to T2 and correlations with clinical variables such as BMI or the maximal reached heart rate (HR) (%) of age‐predicted HR.

### 2.2 Participants

Twenty ACP patients and a matched control group were recruited for the study. The inclusion criteria were [1] UICC‐stage four solid tumor diagnosis [2], current chemotherapy treatment [3], age 18 years or older, and [4] signed informed consent. The exclusion criteria included [1] recent immunotherapy or hormone therapy (within the past three weeks) and [2] medical contraindications for HIIT (e.g., bone metastases with fracture risk, ECOG performance status > 2, or acute infection).

### 2.3 Acute Exercise Intervention

The exercise intervention was a single 20‐min HIIT session (see Figure S1). Exercise intensity was primarily regulated via the rate of perceived exertion (RPE) scale to enable individualized and clinically safe adjustment in a heterogeneous ACP population, with priority given to achieving the RPE target whenever discrepancies with HR or achieved workload occurred. Given expected short‐term fluctuations in patients' physical capacity and differences between individuals, RPE‐based prescription was considered appropriate, whereas heart rate responses and workload relative to body weight were applied as complementary objective criteria. Maximal HR was estimated as [220 minus age (years)]. Resting values were measured before training and one minute after the end of the intervention. The intervention was conducted during outpatient chemotherapy visits, ideally between 10:00 and 12:00 before treatment. For hospitalized patients, it typically took place in the afternoon, aligning with ward routines and equipment availability.

### 2.4 Questionnaires

QoL and fatigue were assessed via the EORTC QLQ‐C30 and EORTC FA‐12 questionnaires. A sociodemographic questionnaire was used to assess education, occupation, family status, weekly physical activity, and exercise interest on a 1–10 scale, with one indicating low interest and 10 indicating high interest. The Mini Nutritional Assessment Questionnaire was used to evaluate nutritional status. All questionnaires were completed during the 60‐min rest period. The objective of these measures was to provide contextual information regarding the symptom burden at baseline. Given the acute nature of the intervention, no post‐exercise follow‐up questionnaires were planned.

### 2.5 Blood Sampling

At T0, T1, and T2, 2.5 mL of blood was collected in an EDTA tube via a peripheral or central catheter and stored at room temperature for flow cytometric analysis within 24 h. Patients were instructed not to eat during the 1‐h recovery period to avoid influencing blood parameters, but drinking water was allowed.

### 2.6 Isolation and Staining of Peripheral Blood Mononuclear Cells (PBMCs)

We used a protocol previously described by Bennstein et al. [27, 36]. The blood was first analyzed via a Sysmex cell counter (model XN‐1000) for differential blood counting and subsequently diluted 1:1 with sterile Phosphate‐Buffered Saline (Dulbeccos's Phosphat Buffered Saline, DPBS). To isolate PBMCs, Ficoll Plaque PLUS (1.077 g/mL) was added for density gradient centrifugation. PBMCs were diluted with DPBS, and remaining erythrocytes were lysed by the addition of erylysis buffer (BD Pharm Lyse, BD Biosciences). Cells were washed with DPBS containing 0.5% BSA (Roth) and 5 mM EDTA (Gibco). Less than 100 μL of each sample was incubated with a custom antibody mixture for 20 min and then washed and centrifuged. The cells were analyzed via FACS on a cytoflex (Beckman Coulter) within 24 h.

### 2.7 Analysis of Flow Cytometry Data and Gating Strategy

**FIGURE 2:**
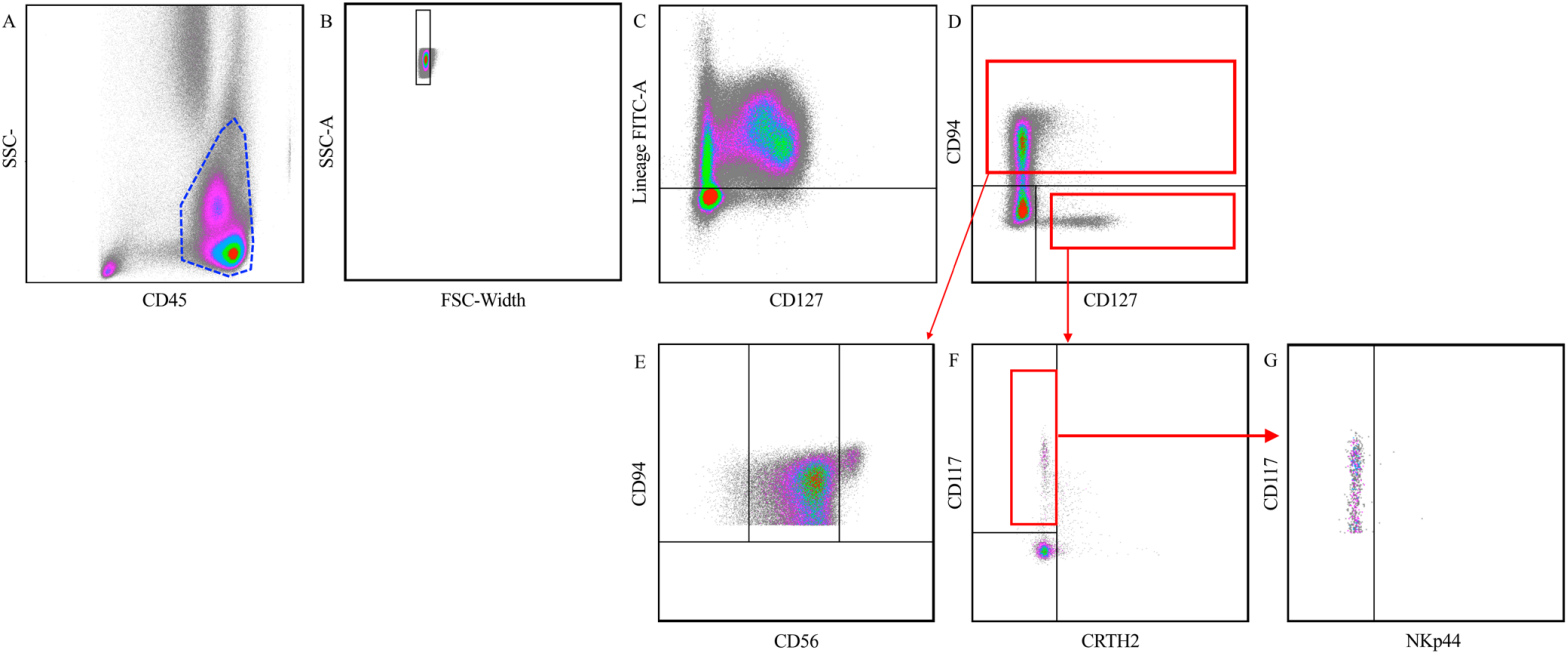
Gating strategy for flow cytometry of peripheral blood mononuclear cells. (A) Selection of CD45^+^ lymphocytes, (B) Gating of single cells, (C) Excluding lineage positive cells, (D) Gating on NK cells (CD94^+^) and cILCs (CD127^+^), (E) Distinguishing NK cells (CD94^+^, CD56^+^) on the basis of CD56 expression into the subgroups of CD56^bright^ (CD94^+^, CD56^++^) and CD56^dim^ (CD94^+^, CD56^+^) NK cells, (F) Dividing cILCs (CD94^−^, CD127^+^) into their subgroups cILC1 (CD117^−^, CRTH2^−^), cILC2 (CD117^+/−^, CRTH2^+^) and cILC3 neutral (CD117^+^, CRTH2^−^), (G) Which are subsequently divided again by their NKp44 expression into NKp44^−^cILC3 and NKp44^+^cILC3.

First, CD45+ lymphocytes were selected (Figure 2A,B) and lineage‐positive cells were excluded (Figure 2C). NK cells were identified via a CD94 antibody, and cILCs were selected with CD127 (Figure 2D). NK cells were categorized into CD56dim and CD56bright subsets on the basis of CD56 expression (Figure 2E). cILCs were grouped into cILC1 (CD117‐, CRTH2‐), cILC2 (CD117+/−, CRTH2+), and cILC3 neutral (CD117+, CRTH2–) (Figure 2F). cILC3 neutral cells were further subdivided into NKp44+ILC3 and NKp44−cILC3 on the basis of NKp44 expression (Figure 2G). The final cell counts were determined via the following formula:

$$\begin{array}{c} \frac{\mathrm{abs}.\text{cell count}\left(\text{flow cytometry}\right)}{\mathrm{abs}.\;\text{lymphocytes}\;\left(\text{flow cytometry}\right)}\\ \times \text{lymphocyte cell count}\;\left(\text{Sysmex},x10^{3}/\mathrm{\mu L}\right). \end{array}$$

### 2.8 Statistical Analysis

Flow cytometry data were analyzed via Kaluza software (Beckman Coulter, version 2.2). Nonparametric tests were used because of the small sample size and mainly nonnormal distribution. The Friedman test was used to evaluate changes within groups from T0 to T1 and T1 to T2. Pairwise discrimination revealed statistical differences. Percent changes from T0 to T1 and from T1 to T2 between groups were analyzed with the Mann–Whitney U test. Spearman's rank correlation coefficient ρ was used to detect correlations between immune response, age, BMI, and the maximum achieved HR (%) of the age‐predicted HR. Correlation analyses were conducted to explore associations between physiological exercise parameters and immune outcomes. HR was used as a surrogate of relative exercise intensity and sympathetic activation, which is known to influence acute immune cell mobilization. BMI was included as an indicator of body composition and metabolic status, both of which may affect baseline immune function as well as the adaptive immune response to exercise. The correlation strength was categorized as weak (Spearman's Rho ρ = 0.10–0.29), moderate (Spearman's Rho ρ = 0.30–0.49), or strong (Spearman's Rho ρ > 0.50). Statistical significance was set at *p* < 0.05, with analysis performed via IBM SPSS version 28.0.1.1. All the graphs were generated via Graph Pad Prism Version 10.4.1 (532).

## 3 Results

### 3.1 Participant Characteristics

**FIGURE 3:**
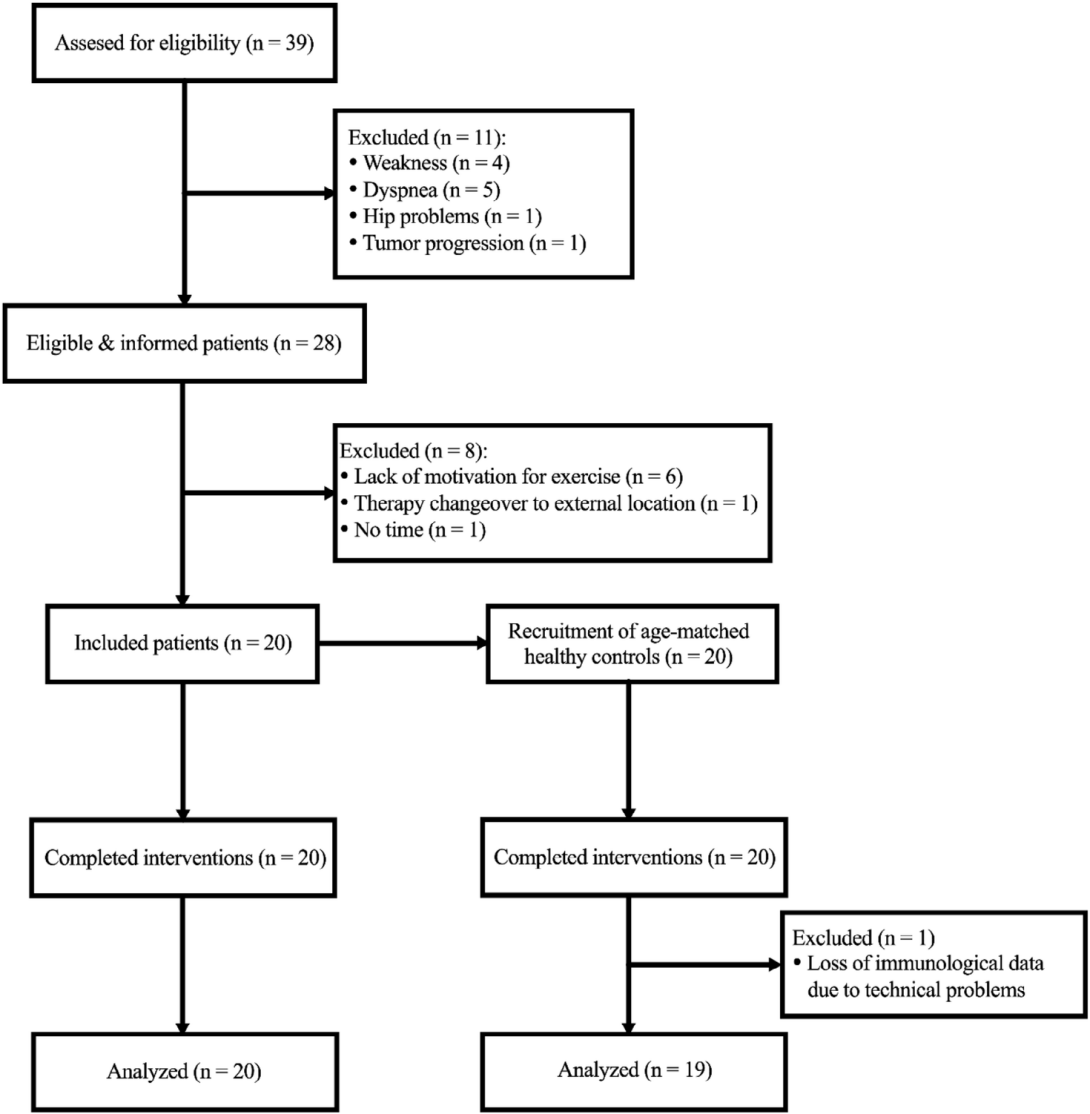
Flowchart of screening and recruitment of patients and reasons for exclusion.

Among the 28 eligible patients, 20 (71%) provided written consent to participate. All included patients were diagnosed with advanced disease and had a UICC stage IV solid tumor at the time of study inclusion. Pancreatic cancer represented the majority of diagnoses. The characteristics of the participants in both the PG and the matched HG are shown in Table 1. One HG participant was excluded from the analysis because of missing immunological data after technical issues. Patient matching was not excluded as it was within two standard deviations in terms of weight and age and reflected the median sporting interest of the patient population (Figure 3).

**TABLE 1:** Patient and subject characteristics.

	Patient group			Control group		
	*N* (%)	Mean (SD)	Median (range)	*N* (%)	Mean (SD)	Median (range)
Gender						
Male	12 (60)			11 (58)		
Female	8 (40)			8 (42)		
Age (years)	20	59.8 ± 10.3	62.5 (31–74)	19	60.7 ± 10.6	61 (29–78)
BMI (kg/m^2^)	20	23.2 ± 4.2	23.1 (17.3–34.9)	19	24.7 ± 2.9	23.9 (20.6–30.5)
Exercise interest						
Before diagnosis	20	7 ± 3	8 (1–10)	0		
Current	20	6 ± 2	7 (1–10)	19	7 ± 2	8 (3–10)
Nutritional status (MNA Questionnaire)						
Normal nutritional status	6 (32)			18 (95)		
Risk for malnutrition	10 (53)			1 (5)		
Malnutrition	3 (15)			0 (0)		
Diagnosis						
Pancreatic carcinoma	14 (70)					
Cholangiocellular carcinoma	2 (10)					
Small cell lung carcinoma	1 (5)					
Leiomyosarcoma	1 (5)					
Gastric carcinoma	1 (5)					
Hemangioendothelioma	1 (5)					
Health related QoL (EORTC QLQ‐C30)	20	53.3 ± 18.4	54.2 (16.7–83.3)	19	86.4 ± 12.5	83.3 (58.3–100)
Fatigue (EORTC FA‐12)						
Physical fatigue	20	36.7 ± 23.3	33.3 (0–80)	19	12.6 ± 12.8	6.7 (0–40)
Emotional fatigue	20	11.1 ± 18.6	0 (0–66.7)	19	2.3 ± 6.8	0 (0–22.2)
Cognitive fatigue	20	26.7 ± 24.7	22.2 (0–77.8)	19	6.4 ± 9.1	0 (0–33.3)
Chemotherapy cycles	19^a^	17 ± 15	14 (3–72)			
Chemotherapy						
Gemcitabin‐based	9 (45)					
Doxorubicin‐based	2 (10)					
5‐FU based	6 (30)					
Others	3 (15)					

*Note:* The data are reported as the means (SDs) and medians (ranges) or counts (*n*). The questionnaires on health‐related QoL and fatigue were assessed via the EORTC QLQ‐C30 (range 0–100, where high scores represent a high quality of life or fatigue). Interest in exercise was assessed via a numerical rating scale ranging from 1 to 10 with 1 = low interest and 10 = high interest.

Abbreviations: 5‐FU, 5‐fluorouracil; BMI, body mass index; MNA, mini nutritional assessment; *n*, number of participants; QoL, quality of life; SD, standard deviation; UICC, union for international cancer control.

^a^ One patient's chemotherapy cycle was undocumented.

### 3.2 Exercise Characteristics

HR during different intervention phases was greater in the HG (see Table 2). Both groups rated the HIIT with 16 ± 1 on the RPE scale. In the HG, the wattage had to be set twice as high as that in the PG to achieve the same load on the RPE scale. In the PG, five participants met both 85% max HR and the prescribed RPE of 15–17, whereas in the HG, 14 participants achieved both thresholds.

**TABLE 2:** Characteristics of the HIIT intervention.

	Patient group			Control group		
	*N* (%)	Mean (SD)	Median (range)	*N* (%)	Mean (SD)	Median (range)
HR (bpm)						
HIIT	20	117 ± 17	112 (91–150)	18^a^	130 ± 22	132 (86–166)
Warm‐up	20	101 ± 17	98 (83–134)	18^a^	93 ± 16	95 (59–115)
Cool‐down	20	110 ± 17	104 (91–152)	18^a^	116 ± 21	116 (71–148)
Total	20	112 ± 18	106 (90–144)	18^a^	116 ± 19	115 (75–145)
Maximal reached HR % from age‐predicted maximal HR	20	91 ± 12	89 (71–117)	18^a^	107 ± 15	108 (74–135)
RPE HIIT	20	16 ± 1	16 (15–18)	18^a^	16 ± 1	16 (15–18)
Reached HR and RPE target values						
No	15 (75)			4 (22)		
Yes	5 (25)			14 (78)		
Workload during HIIT (Watt)	20	53.8 ± 19.4	50 (25–100)	19	93.7 ± 23.1	93.5 (60–140)
RPM (U/min)						
HIIT	20	67.1 ± 15.4	64.5 (27–86)	19	95.7 ± 16.8	95.4 (65–122)
Warm‐up	20	55.9 ± 13.8	55.5 (28–85)	19	71.8 ± 16.1	70.9 (42–108)
Cool‐down	20	62.9 ± 15.9	61.3 (29–92.5)	19	85.5 ± 15.8	86.8 (52–121)
Total	20	63 ± 14.8	59.5 (28–87)	19	86.7 ± 15.4	88.3 (55–113)
Adverse events						
No	20 (100)			19 (100)		

*Note:* The data are reported as the means (SDs) and medians (ranges) or counts (*n*).

Abbreviations: bpm, beats per minute; HIIT, high‐intensity interval training; HR, heart rate; RPE, rate of perceived exertion on the RPE scale; RPM, revolutions per minute.

^a^ Heart rate record missing for one subject, though it was checked during intervention and met requirements.

### 3.3 Mobilization of NK Cells and cILCs

#### 3.3.1 Immune Cell Response Within Groups

**FIGURE 4:**
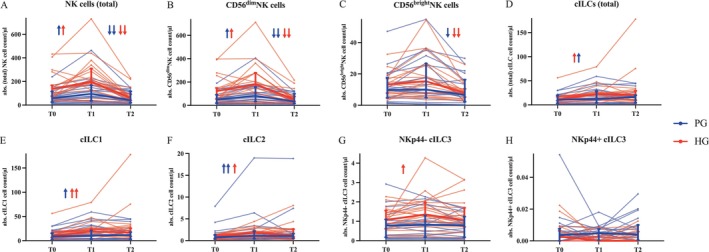
Absolute cell counts/μL at the three sampling points T0, T1 and T2. NK cells (A) and their subpopulations CD56^dim^ (B) and CD56^bright^ (C) NK cells, total number of cILCs (D) and their subpopulations cILC1 (E), cILC2 (F), NKp44^−^cILC3 (G) and NKp44^+^ILC3 (H) of PG (*n* = 20) and HG (*n* = 19), represented as individual data (thin lines) and median values (thick lines) with interquartile ranges (PG = blue, HG = red). T0 = preexercise, T1 = immediately post‐exercise, T2 = one hour post‐exercise after recovery. ↑/↓ = *p* < 0.05; ↑↑/↓↓ = *p* < 0.001 analyzed with the Friedman test.

NK cells (Figure 4A) increased by a median of 50.23% in the PG and 38.38% in the HG from T0 to T1 (PG: *p* = 0.001; HG: p = 0.045) and decreased by 43.29% (PG) and 68.6% (HG) during recovery (PG: *p* < 0.001; HG: *p* < 0.001). CD56dimNK cells (Figure 4B) increased significantly from T0 to T1 (PG: *p* = 0.001; HG: *p* = 0.045) and decreased from T1 to T2 (PG: *p* < 0.001; HG: *p* < 0.001). For CD56brightNK cells (Figure 4C), a significant decrease was observed in both groups during the recovery period (PG: *p* = 0.005; HG: *p* < 0.001). Total cILCs (Figure 4D) were mobilized significantly from T0 to T1 (PG: *p* = 0.005; HG: *p* = 0.001) and increased by a median of 21.49% in PG and 55.49% in HG, with no changes from T1 to T2. cILC1 (Figure 4E) increased in both groups from time point T0 to T1 (PG: *p* = 0.008; HG: *p* < 0.001), but no significant changes were observed during the recovery period. cILC2 (Figure 4F) increased in both groups immediately after the exercise intervention (T0‐T1 PG: *p* < 0.001; HG: *p* = 0.001), with no changes from T1 to T2. Following the exercise intervention, NKp44−cILC3 (Figure 4G) increased significantly only in the HG (p = 0.045), whereas no significant changes were observed in the PG. NKp44+cILC3 (Figure 4H) were found in both groups, but no changes in the cell count were observed at any time point. The underlying cell numbers and changes can be found in Files S3 and S4.

#### 3.3.2 Immune Cell Response Between Groups

**FIGURE 5:**
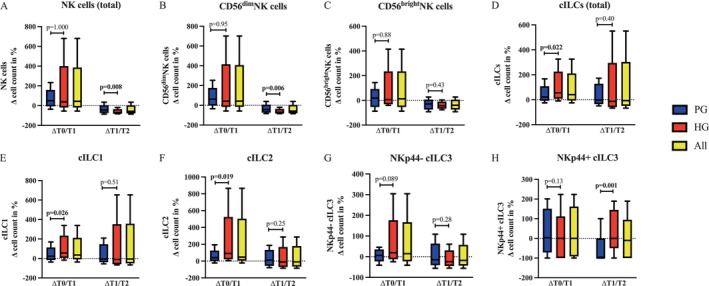
Total percentage changes in cell counts from T0 to T1 and T1 to T2 presented as boxplots with medians, interquartile ranges and minimums to maxima. The boxplots illustrate the percent changes in the cell count, with the median as the marked line, the box as the interquartile range and the whiskers representing the minimum to maximum. The blue boxplots represent the PG, the red boxplots represent the HG, and the yellow boxplots represent both groups. The *p* values indicate significant differences between the PG and HG at T0–T1 and T1–T2, with significant results in bold analyzed with the Mann–Whitney U Test.

Group comparisons revealed no differences in the mobilization of NK cells, NK subgroups, or cILC3 from T0 to T1. For total cILCs (*p* = 0.022; HG: 55.49% (−9.50 to 325.57); PG: 21.49% (−24.89 to 167.88)), cILC1 (*p* = 0.026; HG: 56.15% (−16.74 to 338.60); PG: 23.54% (−36.02 to 169.89)), and cILC2 (*p* = 0.019; HG: 90.25% (5.55 to 865.94); PG: 40.59% (−22.93 to 193.8)), we observed a significantly greater increase with exercise in the HG. The group comparison between T1 and T2 revealed no significant differences in CD56^bright^NK cells, total cILCs, cILC1, cILC2, or NKp44 ^−^ cILC3, whereas total NK cells (*p* = 0.008; HG: −68.6% (−82.79 to −18.37), PG: −43.29% (−85.13 to 35.63)) and CD56^dim^NK cells (*p* = 0.006; HG: −69.71% (−84.75 to −20.13); PG: −43.97% (−84.09 to 38.64)) decreased significantly more in the HG. Furthermore, a significant difference in NKp44 ^+^ cILC3 lymphocytes (*p* = 0.001) was observed from T1 to T2; however, when the HG remained constant (0% (−100 to 189.42)), a decrease was detected in the PG (−100% (−100 to 100)) (Figure 5).

### 3.4 Correlation Analyses

#### 3.4.1 Correlations of Immune Cell Mobilization With Age, BMI, and HR in Both Groups

Significant correlations were identified between immune cell mobilization, age, and the maximal reached HR (%) of the age‐predicted HR and BMI in both groups (Table 3). Younger age correlated with increased CD56^bright^NK cells in the PG (*p* = 0.003). No correlations were found between age, and immune cell mobilization in the HG. A higher BMI correlated with increasing cILC2 (*p* = 0.027) in the HG, whereas no correlations were observed for BMI in the PG. Significant correlations with the maximal reached HR were found in both groups: higher HR correlated with increased total NK cells (PG: *p* = 0.019; HG: *p* < 0.001), CD56^bright^NK cells (PG: *p* = 0.021; HG: *p* = 0.001), and CD56^dim^NK cells (PG: *p* = 0.044; HG: *p* < 0.001) from T0 to T1. Additionally, in the HG, higher maximal HR correlated with increased NKp44 ^−^ cILC3 (*p* = 0.004).

**TABLE 3:** Correlation analyses for percentage changes in immune cell mobilization and clinical data.

	NK cells total (∆%) T0/T1	CD56^dim^ NK cells (∆%) T0/T1	CD56^bright^ NK cells (∆%) T0/T1	cILCs total (∆%) T0/T1	cILC1 (∆%) T0/T1	cILC2 (∆%) T0/T1	NKp44^−^cILC3 (∆%) T0/T1	NKp44^+^ cILC3 (∆%) T0/T1
Age (PG)	−0.409	−0.388	−0.628**	−0.376	−0.431	−0.176	−0.100	−0.365
Age (HG)	−0.085	−0.061	−0.174	0.048	0.031	0.360	−0.001	0.179
BMI (kg/m^2^) (PG)	−0.259	−0.284	−0.170	−0.201	−0.250	−0.296	−0.047	−0.028
BMI (kg/m^2^) (HG)	0.047	0.109	−0.316	0.058	0.005	0.505*	−0.150	0.072
Maximal reached HR (%) of age‐predicted HR (PG)	0.518*	0.454*	0.512*	0.082	−0.032	0.209	0.178	0.308
Maximal reached HR (%) of age‐predicted HR (HG)	0.741**	0.716**	0.704**	0.394	0.416	0.000	0.649**	0.223

*Note:* Spearman's rank correlation coefficient (0.10–0.29 = weak correlation, 0.30–0.49 = moderate correlation, > 0.50 = strong correlation), *p < 0.05, **p < 0.01, *n* = 20 for PG and *n* = 19 for HG.

### 3.5 Analysis of CD16 Expression on CD56^dim^NK Cells

**FIGURE 6:**
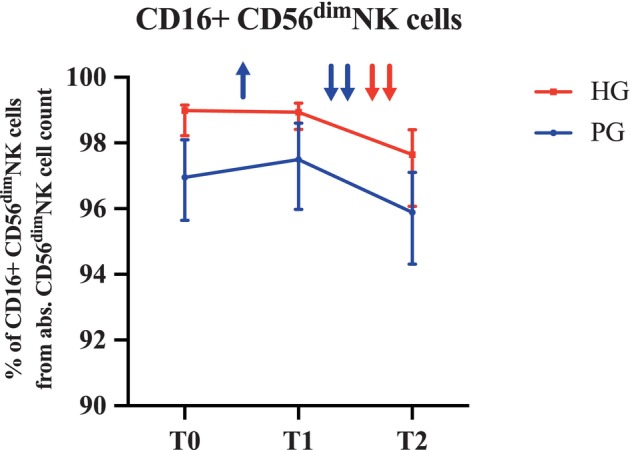
Median percentages and interquartile ranges for CD16^+^CD56^dim^NK cells among all CD56^dim^NK cells at the individual time points. T0 = preexercise, T1 = immediately post‐exercise, T2 = one‐hour post‐exercise after recovery in the different groups: PG (*n* = 20), HG (*n* = 19). ↑/↓ = *p* < 0.05; ↑↑/↓↓ = *p* < 0.001 analyzed with the Friedman test.

From T0 to T1, a significant increase in the percentage of CD16 ^+^ CD56^dim^NK cells among total CD56^dim^NK cells was observed only in the PG (*p* = 0.022), but not in the HG, followed by a significant decrease during recovery (T1 to T2) in both groups (PG: *p* < 0.001; HG: *p* < 0.001) (Figure 6).

## 4 Discussion

This study investigated the effects of a single acute HIIT exercise session on the mobilization of NK cells and cILCs, including their respective subpopulations, in ACP and healthy controls. We observed significant post‐exercise increases in total NK cells, CD56^dim^NK cells, total cILCs, and the cILC1 and cILC2 subgroups in both groups. These findings are in line with previous studies conducted in other oncological populations, such as prostate [22] and breast cancer patients [37], suggesting that acute exercise‐induced immune cell mobilization may also be present in patients receiving palliative cancer care.

Evidence suggests that exercise‐induced elevations in circulating catecholamines [38] may contribute to the rapid redistribution of NK cells, potentially mediated by their high expression of beta‐adrenergic receptors [39]. This response follows an intensity‐dependent dose–response relationship, with higher physiological load associated with greater catecholamine release and NK cell mobilization [40, 41], which may partly explain the stronger responses observed in the healthy controls.

Current evidence in cancer populations suggests that both HIIT and moderate exercise can induce acute immune cell mobilization, with the magnitude of response being primarily driven by exercise intensity rather than training modality [42]. Nevertheless, HIIT has been shown to reliably elicit rapid increases in circulating NK cells in cancer patients, likely due to its high‐intensity nature and associated catecholamine‐mediated sympathetic activation, making it a time‐efficient stimulus for pronounced acute immunological responses. However, clear superiority over other exercise forms has not been established, as recent evidence demonstrates comparable acute NK cell responses following continuous moderate‐intensity and work‐matched HIIT exercise in metastatic cancer patients undergoing chemotherapy, suggesting similar short‐term immunological effects when total work and relative intensity are matched [43].

Given the established role of NK cells in immune surveillance and tumor cell recognition [9], these acute exercise‐induced changes may be relevant in ACP. However, whether such transient increases translate into enhanced tumor infiltration [44], sustained immune modulation, or clinically meaningful outcomes cannot be inferred from the present data and warrants further investigation.

The lack of a significant post‐exercise increase in CD56^bright^NK cells, in contrast to CD56^dim^NK cells, has also been reported in other studies in healthy populations [45]. Previous work suggests that the more pronounced mobilization of cytotoxic CD56^dim^NK cells in contrast to CD56^bright^NK cells may reflect exercise‐induced changes in NK cell cytotoxicity [46], although direct evidence remains limited.

In addition, we observed significant post‐exercise increases in total cILCs and their subgroups cILC1 and cILC2 in both groups. While such responses have been reported previously in healthy subjects [47] and adolescent and adult cancer populations [48], this is the first demonstration of ACP receiving palliative care.

The decline in NK cells and their subsets CD56^dim^ and CD56^bright^NK cells from T1 to T2 may reflect the so‐called open window effect. Peake et al. [49] reported a rapid post‐exercise decrease in lymphocytes, which typically reaches its lowest level approximately 30 min after exercise and returns to baseline within 4–6 h. Although lymphocyte apoptosis was previously suggested [50], this hypothesis has not been confirmed [51, 52]. Current evidence indicates that transient reductions in circulating NK cells are more likely due to redistribution, potentially involving migration into peripheral tissues under the influence of glucocorticoids [53].

In contrast, no significant decrease in cILCs was observed from T1 to T2. Given the limited understanding of cILC dynamics in response to exercise, further research is needed to clarify the plateau observed for cILC1 and cILC2, as well as the decline in NKp44 ^−^ cILC3 in both study populations. These findings may suggest that cILCs are less affected by the open window effect, while the decrease in NKp44 ^−^ cILC3 could reflect tissue redistribution and subsequent maturation into other subsets [54]. Notably, differences between groups were observed in NKp44 ^+^ cILC3: HG increased from T1 to T2, whereas the PG decreased over the same period. Further investigations are needed to better understand the behavior of NKp44 ^+^ cILC3.

Significant differences between the two groups included stronger mobilization of cILCs, cILC1, and cILC2 with exercise in the HG. Given the limited number of studies on the adaptation of cILCs to exercise in both healthy individuals and ACP, this observation warrants further investigation. The greater decrease in total NK and CD56^dim^NK cells from T1 to T2 in the HG, along with the generally lower cell numbers in the PG, may reflect the higher average HR, wattage, and cardiovascular stress in the HG. Additionally, lymphotoxic chemotherapy and cancer‐related immunological dysfunctions, such as lymphopenia, could contribute to lower cell counts and variability in some patients [55], although comparable adaptation phenomena were shown in both study groups.

We observed a significant increase in the percentage of CD16 ^+^ CD56^dim^NK cells from T0 to T1 in the PG, followed by a significant decrease from T1 to T2 in both groups. These findings suggest that a single exercise bout can change CD16 expression on CD56^dim^NK cells, which is a known activator of NK cell cytotoxicity. While this upregulation of CD16 suggests a potential functional relevance of exercise‐induced NK cell redistribution, it should be interpreted in light of the lack of direct functional immune assessments, which will partially be addressed in future analyses using collected plasma.

Several immunological outcomes exhibited wide interquartile ranges, reflecting substantial interindividual variability. This variability likely reflects differences in baseline physical condition, acute health status, and ongoing supportive or anticancer therapies, as well as natural biological variation in immune parameters.

Younger age correlated with an increase in CD56^bright^NK cell counts from T0 to T1 in the PG. Both Solana et al. and Sellami et al. [56, 57] reported a tendency for the number of CD56^bright^NK cells to decrease with age in healthy individuals, which could also influence the mobilization of this subgroup in ACP. A positive correlation was observed between higher BMI and increased mobilization of cILC2 in the HG. Although data on the effects of BMI on cILCs are lacking, Viel et al. [58] showed that the number of NK cells correlates with BMI regardless of exercise type, suggesting that cILCs might behave similarly. Additionally, higher HRs were correlated with increases in total NK cells, CD56^dim^ , and CD56^bright^NK cells in both groups, and with NKp44 ^−^ cILC3 in the HG. Koivula et al. [37] were able to show in cancer patients, as did Neves et al. [59] in healthy individuals, that higher training intensity is associated with greater immune cell mobilization, which is consistent with our findings, although, as noted above, differences in disease stage and treatment should also be considered in ACP. These associations should be interpreted as correlational rather than causal.

### 4.1 Study Strengths and Limitations

This study has several limitations. A notable aspect of the present cohort is the high percentage of pancreatic cancer diagnoses. Pancreatic cancer is characterized by a highly immunosuppressive tumor microenvironment, including increased levels of myeloid‐derived suppressor cells and regulatory T cells [60], as well as impaired NK cell function [61]. These tumor‐specific immunological features may influence both baseline immune cell distributions and their responsiveness to exercise. Caution is warranted when extrapolating the findings to other tumor entities. Another limitation of the present study is therefore the lack of functional immune assays, such as NK cell cytotoxicity or cytotoxic T lymphocyte activity, which limits the clinical relevance of the observed cell mobilization. The absence of a non‐exercise control group prevents distinguishing exercise‐specific effects from other potential influences. Exercise intensity was defined using RPE to reflect subjective perceived exertion in a clinically heterogeneous population with limited muscular strength. This does not ensure equivalence in absolute physiological strain between groups, and between‐group differences should therefore be interpreted accordingly. Due to the inclusion of a patient cohort with ECOG 0–2, the present findings cannot be generalized to the broader population of ACP, particularly those with poorer performance status. The intervention timing could have been standardized to avoid circadian effects, which may have influenced immune cell counts at baseline. The potential influence of ongoing systemic therapies should also be considered when interpreting the present findings. Chemotherapeutic agents commonly used in ACP, such as gemcitabine and 5‐fluorouracil, are known to exert myelosuppressive and lymphotoxic effects, which may affect both baseline immune cell counts and their capacity for mobilization. As patients in this study were at different stages of treatment and received heterogeneous regimens, the timing and type of chemotherapy may have contributed to the observed interindividual variability. Due to the limited sample size, these factors could not be systematically controlled for in the statistical analysis. A formal adjustment for multiple comparisons was not applied, which increases the risk of type I error and should be considered when interpreting the results. Additionally, food intake was restricted during the testing period, but preintervention meals were uncontrolled. Despite these limitations, this study provides valuable insights into the effects of an acute exercise bout in ACP and enables comparisons with a rigorously matched control group, laying the groundwork for future research in larger settings.

## 5 Conclusion

Our study revealed that the immune response to an acute HIIT intervention is comparable between a group of ACP and a matched control group. The mobilization of cytotoxic immune cells suggests that acute high‐intensity exercise interventions may support immune function in palliative care. While the present study concentrated on acute immunological changes subsequent to a single exercise session, potential clinical benefits, such as reductions in fatigue or improved chemotherapy tolerance, are more likely to result from regular and sustained training interventions. The molecular mechanisms underlying exercise‐induced immune cell redistribution are not yet fully understood. Advanced proteomics technologies offer promising opportunities to address this gap, as mass spectrometry‐based approaches enable comprehensive quantification of protein‐level functional states and signaling pathway activation within immune cell subsets [62]. Such approaches may help to validate catecholamine‐driven mobilization mechanisms and further characterize the functional capacity of redistributed NK cells. In addition, emerging high‐dimensional single‐cell and multi‐omics approaches allow for a more detailed analysis of immune cell heterogeneity and dynamic state transitions. The integration of these technologies may provide deeper insights into the regulation, activation status, and functional adaptation of NK cells and other immune populations following acute exercise [63]. In summary, future research should explore long‐term effects, clinical outcomes (e.g., infection rates or treatment tolerance and efficiency), and mechanisms of immune cell redistribution to strengthen the clinical relevance of exercise.

## Author Contributions

**Maria Rosa Salvador Comino:** supervision, writing – review and editing, resources, methodology. **Suzan Botzenhardt:** writing – review and editing, supervision, data curation, investigation. **Sabrina B. Bennstein:** writing – review and editing, data curation, methodology, formal analysis, conceptualization, software, supervision. **Mitra Tewes:** writing – review and editing, funding acquisition, methodology, supervision, project administration, resources. **Miriam Götte:** funding acquisition, supervision, writing – review and editing, methodology, conceptualization, writing – original draft, project administration, resources, software, data curation. **Isabella Deppe:** writing – review and editing, software, methodology, conceptualization, investigation. **Ronja Beller:** supervision, writing – review and editing, methodology, software, conceptualization, data curation. **Nico De Lazzari:** investigation, writing – review and editing, supervision, software, resources. **Fabian Kiehl:** investigation, conceptualization, methodology, formal analysis, data curation, visualization, writing – original draft, writing – review and editing, software, resources, project administration.

## Funding

We acknowledge support from the Open Access Publication Fund of the University Duisburg‐Essen, Germany. We thank the Brigitte und Dr. Konstanze Wegener‐Stiftung for funding.

## Ethics Statement

All participants provided written informed consent in accordance with the Declaration of Helsinki. The protocol was approved by the Ethics Committee of University Hospital Essen, Germany (approval number: 19‐8789‐BO) 13.10.2022.

## Consent

All the authors read and approved the final manuscript for publication.

## Conflicts of Interest

The authors declare no conflicts of interest.

## Supporting information


**File S1:** Schematic overview of the HIIT intervention and phase‐specific target values.
**File S2:** Flow cytometry analysis.
**File S3:** Median absolute cell counts and absolute cell count changes of NK cells, cILCs, and their respective subpopulations for the patient group.
**File S4:** Median absolute cell counts and absolut cell count changes of NK cells, cILCs, and their respective subpopulations for the healthy control group.

## Data Availability

The data presented here can be obtained from the corresponding author upon request.
