# Supplementary material for: High‐Intensity Interval Training Boosts Immune Cells in Advanced Cancer Patients and Healthy Controls: Implications for Cancer Care
Source: Cancer Med. 2026 Jun 3;15(6):e71977. doi: 10.1002/cam4.71977 (PMC13240137; doi:10.1002/cam4.71977)
Supplement: Supplementary file 1 — File S1: Schematic overview of the HIIT intervention and phase‐specific target values. File S2: Flow cytometry analysis. File S3: Median absolute cell counts and absolute cell count changes of NK cells, cILCs, and their respective subpopulations for the patient group. File S4: Median absolute cell counts and absolut cell count changes of NK cells, cILCs, and their respective subpopulations for the healthy control group. [file CAM4-15-e71977-s001.docx]

**Supplementary Material to: High-Intensity Interval Training Boosts Immune Cells in Advanced Cancer Patients and Healthy Controls: Implications for Cancer Care**

**Supplemental file 1 (S1): Schematic Overview of the HIIT Intervention and Phase-Specific Target Values**

**
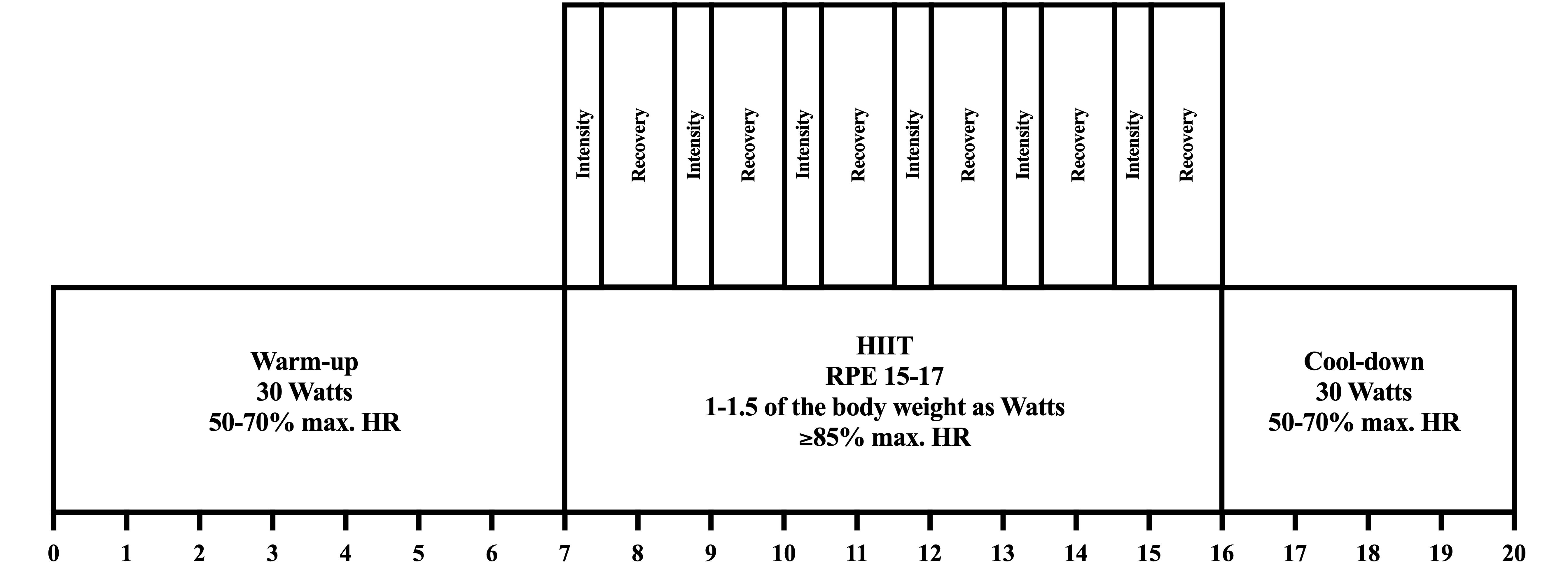
**

**Figure 1:** Schematic illustration of the 20-minute HIIT protocol including warm-up, interval phase, and cool-down, with target intensities and interval structures indicated over time.

Abbreviations: RPE = rate of perceived exertion on the RPE scale, HR = heart rate, HIIT = high intensity interval training

**Supplemental file 2 (S2): Flow cytometry analysis**

As it was described by Bennstein et al, 2019 the isolated cells were stained by using the following FITC conjugated antibodies for the lineage panel: anti-CD1a (HI149), anti-TCRαβ (IP26), anti-TCR𝛾𝛿 (B1), anti-CD123 (6H6), anti-FceR1a (AER-37(CRA-1)), anti-CD235a (HI264) from BioLegend (CA, USA), and anti-CD3 (UCHT1), anti-CD14 (RMO52), anti-CD19 (J3-119) from Beckman Coulter (CA, USA). Also, the following antibodies were further used for analysis: anti-CD94-PE/Cy7 (DX22), anti-CD56 (NCAM)-BV650^TM^ (HCD56), anti-CD117-BV421^TM^ (104D2), anti-CD294 (CRTH2)-PE/Dazzle 594^TM^ (BM16), anti-CD16-BV605^TM^ (3G8), anti-CD45-APC/Cy7 (HI30) all from BioLegend. Anti-CD336 (NKp44)-BV510^TM^(P44-8) from BD (New Jersey, USA), anti-CD127-PC5-PE/Cy5 (R34.34) and anti-CD159a (NKG2A)-APC (Z199) from Beckman Coulter.

**Supplemental file 3 (S3): Median absolute cell counts and absolute cell count changes of NK cells, cILCs, and their respective subpopulations for the patient group**

|  | T0 | T1 | ∆ T0/T1 | T2 | ∆ T1/T2 |
| --- | --- | --- | --- | --- | --- |
| NK cells/µl (abs) | 57.6 (7.37 – 239.35) | 95.05 (8.61 – 464.08) | **29.5 (-19.52 - 224.73) *** | 40.77 (3.98 – 147.38) | **-32.35 (-316.7 - 19.34) **** |
| CD56^dim^NK cells/µl (abs) | 50.56 (6.02 – 190.06) | 81.53 (7.61 – 405.96) | **27.59 (-17.73 - 215.48) *** | 33.45 (3.58 – 124.31) | **-28.57 (-286.19 - 11.81) **** |
| CD56^bright^NK cells/µl (abs) | 9.82 (0.88 – 47.11) | 10.02 (0.22 – 54.81) | 1.82 (-2.03 – 16.3) | 6.6 (0.23 – 29.95) | **-2.68 (-28.48 - 3.48) *** |
| cILC/µl (abs) | 10.53 (1.06 – 30.15) | 12.08 (0.96 – 59.39) | **1.75 (-4.29 - 29.24) *** | 16.44 (1.82 – 44.91) | -0.18 (-15.95 – 14.76) |
| cILC1/µl (abs) | 8.95 (0.83 – 28.63) | 10.27 (0.72 – 56.88) | **1.2 (-4.33 - 28.25) *** | 11.78 (1.05 – 42.70) | -0.31 (-14.18 – 13.42) |
| cILC2/µl (abs) | 0.76 (0.17 – 7.9) | 1.09 (0.17 – 19) | **0.24 (-0.22 - 11.1) *** | 1.06 (0.26 – 18.87) | 0.005 (-4.99 – 4.83) |
| NKp44^-^cILC3/µl (abs) | 0.79 (0.06 – 2.92) | 0.82 (0.06 – 2.27) | 0.02 (-0.66 – 0.6) | 0.79 (0.05 – 1.71) | -0.02 (-1.08 – 0.88) |
| NKp44^+^cILC3/µl (abs) | 0.004 (0 – 0.05) | 0.005 (0 – 0.019) | 0.0002 (-0.04 – 0.01) | 0.004 (0 – 0.03) | 0 (-0.01 – 0.02) |

*p < 0.05, **p <0.001; n = 20

**Supplemental file 4 (S4): Median absolute cell counts and absolut cell count changes of NK cells, cILCs, and their respective subpopulations for the healthy control group**

|  | T0 | T1 | ∆ T0/T1 | T2 | ∆ T1/T2 |
| --- | --- | --- | --- | --- | --- |
| NK cells/µl (abs) | 135.7 (30.72 – 432.52) | 198.84 (27.59 – 731.03) | **52.19 (-155.09 - 331.6) *** | 59.3 (20.4 – 229.58) | **-131.87 (-501.45 - -5.07) **** |
| CD56^dim^NK cells/µl (abs) | 122.41 (20.75 – 397.48) | 180.85 (19.7 – 712.68) | **53.7 (-147.3 - 321.74) *** | 49.8 (14.36 – 215.89) | **-117.14 (-496.79 - -5.34) **** |
| CD56^bright^NK cells/µl (abs) | 13.27 (2.97 – 32.5) | 15.27 (3.41 – 54.43) | 0.81 (-6.24 – 37.34) | 7.93 (2.74 – 26.08) | **6.14 (-41.49 - 0.11) **** |
| cILC/µl (abs) | 11.37 (3.17 – 56.11) | 22.15 (2.87 – 79.16) | **6.06 (-0.3 - 31.51) *** | 20.66 (3.58 – 177.85) | -2.96 (-28.99 – 98.7) |
| cILC1/µl (abs) | 10.06 (2.71 – 53.75) | 18.79 (2.26 – 72.72) | **4.67 (-0.45 - 27.93) *** | 17.02 (2.43 – 166.46) | -1.91 (-26.49 – 93.74) |
| cILC2/µl (abs) | 0.97 (0.2 – 1.54) | 1.78 (0.31 – 4.4) | **0.51 (0.05 - 3.41) *** | 1.24 (0.11 – 8.08) | -0.31 (-2.38 – 3.68) |
| NKp44^-^cILC3/µl (abs) | 1.06 (0.18 – 2.44) | 1.36 (0.23 – 4.27) | **0.26 (-0.47 - 3.22) *** | 1.05 (0.13 – 3.15) | -0.20 (-1.43 – 1.07) |
| NKp44^+^cILC3/µl (abs) | 0.003 (0 – 0.02) | 0 (0 – 0.009) | 0 (-0.02 – 0.006) | 0 (0 – 0.01) | 0 (-0.009 – 0.01) |

*p < 0.05, **p <0.001; n = 19
